# The Clinical Significance of the Magee Equations in Breast Cancer Prognostication

**DOI:** 10.7759/cureus.81528

**Published:** 2025-03-31

**Authors:** Shubhneet Bal, Roopa Kumari

**Affiliations:** 1 Pathology, Danbury Hospital, Danbury, USA; 2 Pathology, Mount Sinai Morningside, New York, USA

**Keywords:** breast cancer, gene expression assay, magee equation, neoadjuvant therapy (nac), oncotype dx

## Abstract

The landscape of breast cancer management has been revolutionized by advancements in molecular diagnostics, yet accessibility and cost remain significant barriers for many patients. Limited-resource settings often face significant challenges in accessing expensive molecular tests, which can impact timely diagnosis and treatment decisions. The Magee equations present a practical, cost‐effective solution that can bridge this gap, ensuring that patients, regardless of their financial or geographic limitations, receive appropriate and timely treatment. Originally developed at the University of Pittsburgh Medical Center, these equations offer a reliable alternative for estimating recurrence scores without the prohibitive costs associated with genomic assays.

## Editorial

A game changer in breast cancer prognostication

Breast cancer is a systemic disease that requires multiple treatment approaches. Surgery and radiation therapy primarily address the tumor at its original site, while the majority of patients also receive some form of systemic therapy. For estrogen receptor (ER)-positive tumors, hormonal therapy is standard, whereas patients with ER-negative tumors usually receive chemotherapy. In some ER-positive cases, chemotherapy may be added depending on additional risk factors. Key factors for choosing systemic therapy include tumor size, lymph node status, patient’s age, histologic grade, Ki-67 index, and overall receptor profile [1]. In cases of advanced-stage disease with multiple positive lymph nodes, systemic therapy is often recommended. In early-stage or node-negative disease, the receptor status is crucial in deciding the treatment approach.

The majority of breast cancers are ER-positive/HER2-negative; nearly all these patients receive hormonal therapy, but only a small subgroup also benefits from chemotherapy. Identifying which ER-positive/HER2-negative tumors gain advantage from chemotherapy while vigilantly keeping a check not to overtreat the patient is a central question for the multidisciplinary team. In recent decades, advancements in genomics and tumor profiling have significantly improved our understanding of breast cancer biology and pathophysiology. By utilizing a prospectively designed gene expression assay in combination with an algorithm to determine recurrence scores (RSs) and prognostic signatures, we have effectively quantified the risk of distant recurrence in patients. This method has demonstrated greater accuracy compared to traditional approaches, such as assessing ER protein levels and HER2 status along with other factors, which exhibited only limited predictive value for distant recurrence risk in this study [1,2]. Initially developed as prognostic tools, most gene expression assays were later recognized for their utility in guiding treatment decisions in neoadjuvant settings [3]. As a result, gene expression assays such as Oncotype DX, which provides an RS to guide chemotherapy decisions, and MammaPrint, which classifies tumors into high- or low-risk categories, have gained widespread commercial acceptance. The average cost of an Oncotype DX test was approximately $3,500, based on Medicare reimbursement rates in 2018 [4]. While gene expression profile assays are considered highly cost-effective, as noted by Chandler et al. [4], this is more applicable to countries with ample resources and a higher GDP per capita. In contrast, resource-limited nations may face challenges in adopting these assays. According to Batra et al., physicians in such regions are increasingly turning to alternative prognostic tools like Predict and Predict Plus (UK-based online tools for prognosis), as well as other similar resources [5,6]. Among these alternatives, Magee equations (MEs) have emerged as extensively tested and validated predictive and prognostic tools. Their popularity has grown since the algorithm was first presented at the United States and Canadian Academy of Pathology annual meeting in 2006.

The MEs utilize standard histopathologic and immunohistochemical parameters to predict recurrence risk and potential chemotherapy benefit. Unlike Oncotype DX and other genomic assays - which can be costly and time-consuming - the MEs rely on routinely collected immunohistochemical data, making them widely applicable across diverse clinical settings. Initially derived from just 42 patient samples, the equations’ foundation was fairly narrow; however, as clinicians began ordering more ODX tests for ER-positive breast cancers, the number of collected cases grew substantially to include over 1,000 cases of approval and validation [7]. There are three main versions of the MEs:

ME1: \[ \text{Recurrence Score} = 15.31385 + (\text{Nottingham Score} \times 1.4055) + (\text{ER}_{IHC} \times -0.01924) + (\text{PR}_{IHC} \times -0.02925) \] \[ + \begin{cases} 0, & \text{HER2 negative} \\ 0.77681, & \text{HER2 equivocal} \\ 11.58134, & \text{HER2 positive} \end{cases} + (\text{Tumor Size} \times 0.78677) + (\text{Ki-67 Index} \times 0.13269) \]

ME2: \[ \text{Recurrence Score} = 18.8042 + (\text{Nottingham Score} \times 2.34123) + (\text{ER}_{IHC} \times -0.03749) + (\text{PR}_{IHC} \times -0.03065) \] \[ + \begin{cases} 0, & \text{HER2 negative} \\ 1.82921, & \text{HER2 equivocal} \\ 11.51378, & \text{HER2 positive} \end{cases} + (\text{Tumor Size} \times 0.04267) \]

ME3: \[ \text{Recurrence Score} = 24.30812 + (\text{ER}_{IHC} \times -0.02177) + (\text{PR}_{IHC} \times -0.02884) \] \[ + \begin{cases} 0, & \text{HER2 negative} \\ 1.46495, & \text{HER2 equivocal} \\ 12.75525, & \text{HER2 positive} \end{cases} + (\text{Ki-67} \times 0.18649) \]

ME1 integrates the Nottingham score, tumor size, ER, progesterone receptor (PR), HER2, and Ki-67. ME2 is similar to ME1 but excludes Ki-67. ME3 uses only semiquantitative immunohistochemical results for ER, PR, HER2, and Ki-67. The equations help classify cases into three ODX risk categories: low (<18), intermediate (18-30), and high (≥31). Concordance between the actual RS and the RS predicted by MEs was generally high (>95 certainty) [8], with a two-step discordance (low vs. high or high vs. low) of only 0% to <1% [9]. These equations are made available through a free website (https://path.upmc.edu/onlineTools/mageeequations.html) or an app available on iOS devices [10].

Role in the neoadjuvant setting

In a single-institution retrospective study by Farrugia et al. [10], researchers evaluated the ability of ME3 scores derived from pre-therapy core biopsies to predict pathologic complete response (pCR) in ER-positive, HER2-negative/equivocal breast cancer patients who received neoadjuvant chemotherapy. Out of 237 cases, ME3 scores were grouped into low (<18), intermediate (18 to <31), and high (≥31), yielding pCR rates of 0%, 4%, and 36%, respectively. Patients in the high-score category were 13 times more likely to achieve pCR compared to those with scores below 31. Overall, these findings suggest that a ME3 score ≥31 may help identify which ER-positive, HER2-negative/equivocal tumors are likely to benefit from chemotherapy. A subsequent multi-institutional study showed the validity of MEs beyond their original institutional study. This study involved seven academic and nonacademic centers and analyzed a combined dataset of 166 cases. The findings closely mirrored those of the previously published single-institution study. The observed pCR rates based on ME3 scores were 0% (0 of 64) for ME3 <18, 0% (0 of 46) for ME3 between 18 and 25, 14% (3 of 21) for ME3 >25 but <31, and 40% (14 of 35) for ME3 ≥31 (p < 0.0001) [11].

Cost-effectiveness and accessibility

One of the most compelling advantages of the MEs is their ability to mitigate healthcare costs. The Magee Decision Algorithm, which integrates these equations with the mitotic activity score, has been demonstrated to help identify patients who may safely forgo Oncotype DX testing, potentially saving an estimated $300,000 per 100 clinical requests. In resource-limited settings, where expensive molecular testing is often not an option, the MEs provide an invaluable alternative that not only conserves financial resources but also expedites treatment planning.

Immediate availability of these results allows for more rapid clinical decision-making. This is particularly beneficial in settings where delays in genomic testing can significantly impact patient outcomes. Oncologists can now initiate treatment sooner, reducing patient anxiety and potentially improving survival rates.

Embracing precision medicine in everyday practice

Having experienced the challenges of providing advanced oncologic care in low-resource settings, we recognize that access to expensive molecular tests like Oncotype DX is often limited. The MEs offer a practical and cost-effective approach by utilizing readily available histopathologic data to guide treatment decisions. Integrating these equations into routine pathology workflows ensures that patients in resource-limited environments receive personalized, evidence-based care without the additional financial burden.

The movement toward precision medicine necessitates tools that are both effective and accessible. The MEs exemplify this principle by providing an accurate, cost-free method for personalized treatment planning. Their integration into daily practice enhances risk assessment, optimizes chemotherapy recommendations, and reduces unnecessary healthcare expenditures.

Conclusions

The MEs represent a significant advancement in breast cancer management. Their ability to provide reliable recurrence risk estimates, support informed treatment decisions, and serve as a cost-effective alternative to genomic testing makes them an essential component of contemporary oncology practice. As their use becomes more widespread, these equations will play a pivotal role in improving breast cancer care globally, ensuring that patients receive the best possible treatment regardless of their geographic or financial constraints.
